# Mathematical model of the spread of COVID-19 in Plateau State, Nigeria

**DOI:** 10.1186/s42787-022-00144-z

**Published:** 2022-04-28

**Authors:** O. Adedire, Joel N. Ndam

**Affiliations:** 1 Department of Mathematics, University of Jos, Jos, Nigeria; 2 Federal College of Forestry, Jos, Plateau State Nigeria

**Keywords:** COVID-19, Coronavirus, Disease, Epidemic, Plateau State, Nigeria, 97M10, 93A30, 81T80, 34C60, 03C30, 00A71

## Abstract

In this research, a mathematical model consisting of non-pharmaceutical control measures is formulated. The developed model helps to examine the transmission of COVID-19 infection in Plateau State, Nigeria, using face masks $c_{f}$ and social distancing $c_{d}$ as control measures. Data used for the simulation of the developed model were obtained from Nigeria Centre for Disease Control which was fitted to the system of ordinary differential equations using nonlinear least squares method. Results at baseline values $c_{f} = 0.1$ and $c_{d} = 0.2$ of control measures indicate 2.3 estimation as basic reproduction number which suggests that COVID-19 in Plateau State tends towards endemic state. However, above about 40% in the use of face masks in the population and corresponding above 50% adherence to social distancing could as well bring down the basic reproduction number to a value below 1 necessary for disease eradication. The results at baseline values further indicate that the peak of the COVID-19 had been reached in less than 250 days from the first detection date after about 476,455 undetected asymptomatic individuals, 92,168 undetected symptomatic individuals and 83,801 detected quarantined individuals have been fully infectious. Therefore, the policymakers in Plateau State have the possibility of eradicating the disease with further strict non-pharmaceutical control measures provided that the present conditions of analysis remain fairly the same.

## Introduction

SARS-CoV-2 is the virus strain of COVID-19 which was first discovered in Wuhan, Hubei China [1]. It is one of the viral infectious diseases which had spread fast across the world. Plateau State, which is one of the thirty six states of Nigeria situated in Africa, detected its first case of the infectious disease on 23 April 2020. However, prior to the detection of the first incidence case, the Plateau State government declared a lockdown on 9 April 2020 which lasted till 15 April 2020 [2, 3].

There has been a considerable spread of COVID-19 in Plateau State among the estimated population of 4,200,442 people [4], despite various non-pharmaceutical control measures put in place to curb the spread of the virus. Consequently, five COVID-19 isolation centres were provided with a total of 217 bed spaces and provision for home-based care (HBC) was made as an alternative strategy whenever the available bed spaces are filled up. The policymakers of Plateau State also provided preventive materials like face masks and hand sanitizers through their Emergency Operations Centres (EOC) to support patients on HBC—which started in July 2020—in order to stop the spread of the disease to family members. Some measures and materials put in place to reduce the spread of the virus include: use of face masks, hand washing, use of infrared thermometers, hand sanitizers, mass media, social distancing, introduction of mobile testing centres for effective detection, isolation of infected individuals and closure of schools, inter-state borders, among others [5–7]. While as of 11 June 2020, the state had tested 2032 persons for COVID-19, out of which 130 were confirmed positive with 3 deaths, 26 persons were on admission at isolation centres, 99 persons discharged and 35 people were of interest at quarantine centres in Pankshin, Qua’an Pan, Heipang and Mangu [5]. However, as of 28 February 2021, 62,317 samples from people have been tested in Plateau State with 8889 confirmed positive cases [8]. Furthermore, a curfew was imposed from 10 p.m. to 4 a.m.; restriction on gatherings of not more than 50 people under strict observance of COVID-19 safety protocols was also announced [9].

The research of Dauda et al. [3] revealed the effect of lockdown, locust of control and state anxiety among residents of Plateau State Nigeria at the time when only three cases of COVID-19 were reported with the aid of an online survey using statistical analysis approach. Few researchers have actually considered the spread of COVID-19 in Nigeria [3, 10–12].

While the research of Iboi et al. [10] focused on transmission of COVID-19 in Lagos, Kano and Federal Capital Territory, the study conducted by Okuonghae and Omame [12] focused on Lagos State only. They both investigated the impact of non-pharmaceutical control measures in their areas of coverage. Dauda et al. [3] on the other hand focused on Plateau State with emphasis on statistical analysis of lockdown, locus of control and state anxiety among residents. Furthermore, while some studies focused on hybrid fractional order, fractional optimal control and stochastic models [13–16], some other recent studies predicted outbreak of coronavirus and assessment of control measures as well as Neyman–Scott point process model for COVID-19 both within and across international borders [17–20]. However, from the foregoing researches and other previously published works [21–27], there has not been any published research to date which has considered a mathematical model approach for the transmission dynamics of the spread of COVID-19 in Plateau State Nigeria.

Different types of numerical methods have been proposed for solving mathematical models. The type of numerical method used for any system of differential equations could largely be dependent on the nature of the model. Alotaibi et al. [28] investigated solution of COVID -19 model, and they concluded that homotopy annoyance and decreased differential change techniques are adequate for solving such model equations. Modified reduced differential transform and homotopy perturbation method have also been used in some other researches [29, 30]. In the research of Mahdy et al. [31], they investigated Rubella ailment disease model using shifted second-order Chebyshev polynomials type which they claimed had not been used before and concluded that the method is efficient and its simple-to-use style makes it suitable for finding convergent solutions to Rubella disease models. Simos [32] solved problems with oscillating solutions with Runge–Kutta–Fehlberg method with phase-lag of order infinity and showed its suitability for such problems. Also, Paul et al. [33] investigated behaviour of Lotka–Volterra prey–predator model using Runge–Kutta–Fehlberg method and Laplace–Adomian decomposition method and presented their effectiveness in solving such problems. The research of Handapangoda et al. [34] used Laguerre Runge–Kutta–Fehlberg method for simulating laser pulse propagation in biological tissue. In this study, the proposed model will be solved with existing numerical method based on Fehlberg fourth–fifth-order Runge–Kutta method. For more details on Runge–Kutta–Fehlberg method and some other suitable numerical methods, readers may check [32–36]

The incentive for this study came from high incidence rate of COVID-19 in Plateau State according to the data obtained from Nigeria Centre for Disease Control (NCDC) as of 28 February 2021. The high incidence rate of the disease could be due to an increase in detection rate which is directly proportional to an increase in number of laboratory tests conducted in Plateau State. There was a remarkable increase in the number of samples tested in Plateau State compared to other states in Nigeria within the period of this study. The total number of samples tested in the laboratory was 62,317 samples, out of which 8889 samples were confirmed positive for COVID-19 as of 28 February 2021. Thus, it is advantageous to measure the impact of non-pharmaceutical interventions on the spread of COVID-19 in Plateau State Nigeria.

In this study, we develop a mathematical model to investigate the effect of non-pharmaceutical interventions on the spread of COVID-19 in Plateau State, Nigeria. It is essential to use non-pharmaceutical control measures since non-pharmaceutical control measures were used throughout the period of this study.

The organization of the remaining parts of this study is as follows: “Methods” section  contains the methods used; “Analysis of model (1)” section  considers the analysis of the developed model. Results and discussion are considered in “Results and discussion” section, and conclusion comes up in “Conclusion” section.

## Methods

### Epidemic data

**Fig. 1:**
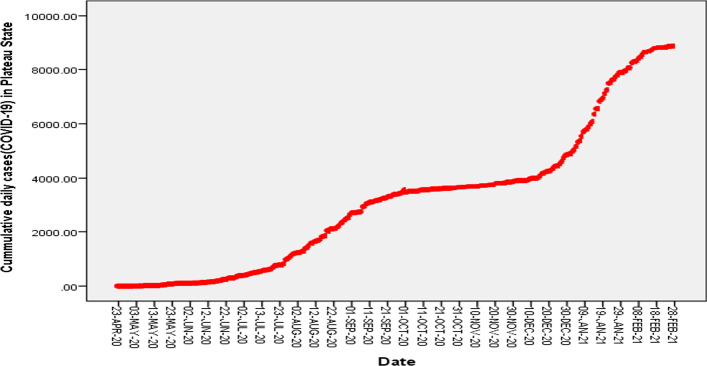
Cumulative daily COVID-19 incidence in Plateau State Nigeria (23 April 2020–28 February 2021)

The epidemic data for this research were obtained from the Nigeria Centre for Disease Control. The centre alerted the Plateau State residents of its first COVID-19 incidence case on 23 April 2020 which prompted the Plateau State government to introduce various non-pharmaceutical measures as control strategies. Laboratories were set up to increase the detection of COVID-19 incidence rate. There was setting up of quarantine centres, sensitization of the public through the media houses, compulsory use of face masks in public places and enforcement of social distancing among residents in the state. Figure 1 shows reported cumulative daily COVID-19 cases from 23 April 2020 to 28 February 2021.

### Model formulation

**Fig. 2:**
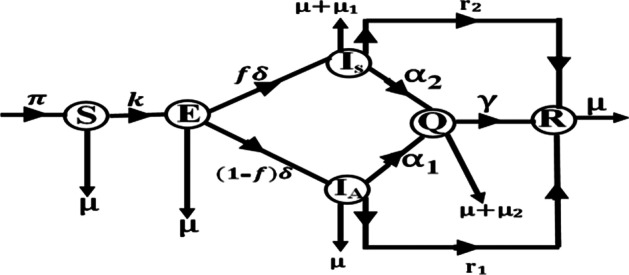
The schematic representation of transmission of COVID-19 infections in Plateau State, Nigeria. $k = \frac{1}{N(t)}(1 - c_{f} )(1 - c_{d} )\left[ {\beta_{E} E(t) + \beta_{{I_{A} }} I_{A} (t) + \beta_{{I_{S} }} I_{S} (t)} \right].$

The formulation of the model for this study is based on human to human transmission of the COVID-19 at a time t. Let the total human population at the time *t* be denoted by $N(t)$ and the population of the susceptible people be $S(t)$. Let the latent population be $E(t)$ and infectious asymptomatic and infectious symptomatic population at time $t$ be $I_{A} (t)$ and $I_{S} (t)$, respectively. While $Q(t)$ represents asymptomatic and symptomatic quarantined population, denote human population that recovered from the COVID-19 by $R(t)$ at time *t* such that $N(t) = S(t) + E(t) + I_{A} (t) + I_{S} (t) + Q(t) + R(t)$. It should be noted that the derivation of the model used the assumption that the exposed class $E(t)$ consists of two sets of people that could transmit the disease and those that could not transmit the disease at latent stage [27]. The schematics representation of the transmission of the COVID-19 is shown in Fig. 2.

This schematic representation of transmission of COVID-19 infection in Fig. 2 is a tool aiding the formulation of the proposed governing model equations for this study. It should be noted that schematic flow display of infection transmission dynamics is essential to infectious disease modelling whenever some details of model formulations are required in the study of biomathematics. This further suggests that the schematic flow representation could be used to check the correctness or otherwise of the governing model equations. It also means that relationship exists between the schematic flow representation of Fig. 2 and system of ordinary differential equations used for this study. For further details on similar studies where schematic flow representation can be found, readers may check [12, 37].

In this study, the spread of COVID-19 in Plateau State Nigeria for the community of people which are distributed homogenously are governed by the following system of differential equations:

1 $$\left. {\begin{array}{*{20}l} {\frac{{{\text{d}}S(t)}}{{{\text{d}}t}} = \pi - \frac{S(t)}{{N(t)}}(1 - c_{f} )(1 - c_{d} )\left[ {\beta_{E} E(t) + \beta_{{I_{A} }} I_{A} (t) + \beta_{{I_{S} }} I_{S} (t)} \right] - \mu S,} \hfill \\ {\frac{{{\text{d}}E(t)}}{{{\text{d}}t}} = \frac{S(t)}{{N(t)}}(1 - c_{f} )(1 - c_{d} )\left[ {\beta_{E} E(t) + \beta_{{I_{A} }} I_{A} (t) + \beta_{{I_{S} }} I_{S} (t)} \right] - f\delta E(t)} \hfill \\ \qquad { - (1 - f)\delta E(t) - \mu E(t),} \hfill \\ {\frac{{{\text{d}}I_{A} (t)}}{{{\text{d}}t}} = (1 - f)\delta E(t) - \alpha_{1} I_{A} (t) - (\mu + \mu_{1} )I_{A} (t) - r_{1} I_{A} (t),} \hfill \\ {\frac{{{\text{d}}I_{S} (t)}}{{{\text{d}}t}} = f\delta E(t) - \alpha_{2} I_{S} (t) - (\mu + \mu_{2} )I_{S} (t) - r_{2} I_{S} (t),} \hfill \\ {\frac{{{\text{d}}Q(t)}}{{{\text{d}}t}} = \alpha_{1} I_{A} (t) + \alpha_{2} I_{S} (t) - \gamma Q(t) - (\mu + \mu_{3} )Q(t),} \hfill \\ {\frac{{{\text{d}}R(t)}}{{{\text{d}}t}} = \gamma Q(t) + r_{1} I_{A} (t) + r_{2} I_{S} (t) - \mu R(t),} \hfill \\ {N(t) \, = \, S(t) \, + \, E(t) \, + \, I_{A} \, (t) \, + \, I_{S} \, (t) \, + \, Q(t) \, + \, R(t).} \hfill \\ \end{array} } \right\}$$

where $\pi$ represents recruitment by birth, $\mu$ and $\mu_{i} (i = 1,2)$ are natural deaths and deaths due to COVID-19, respectively. The parameters $c_{f}$ and $c_{d}$ are the non-pharmaceutical control measures representing the use of face mask and social distancing for $0 \le c_{f} \le 1,0 \le c_{d} \le 1.$

Descriptions of the state vectors and other associated parameters of the system of Eqs. (1) are shown in Tables 1 and 2.

**Table 1:** Description of the state variables for model (1)

State variable	Description
*S*	Susceptible population
*E*	Exposed population consisting of transmitting group and transmitting group with low probability of infection transmission
*I*_*A*_	Undetected asymptomatic infectious population
*I*_*S*_	Undetected symptomatic infectious population
*Q*	Detected quarantined asymptomatic and symptomatic infectious population
*R*	Recovered

**Table 2:** Parameters for model (1) and their descriptions

Parameter	Description
$\beta_{E} ,\beta_{{I_{A} }} ,\beta_{{I_{S} }}$	Rate of infection transmission among the exposed transmitting, asymptomatic infectious and symptomatic infectious groups
$c_{f} ,c_{d}$	Proportion of people that adopt control measures using face masks and practice of social distancing
$f$	Fraction of exposed group that becomes symptomatic infectious undetected group $I_{S}$
$1 - f$	Fraction of exposed group that becomes asymptomatic infectious undetected group $I_{A}$
$\delta$	Rate of progression of exposed group E to fully infectious groups $I_{S}$,$I_{A}$
$\alpha_{1} ,\alpha_{2}$	Rate at which people are quarantined from both asymptomatic and symptomatic infectious groups $I_{A}$,$I_{S} .$
$\gamma ,r_{1} ,r_{2}$	Recovery rates from quarantined, asymptomatic and symptomatic groups, respectively
$\pi ,\mu ,\mu_{i} (i = 1,2)$	Recruitment through births, natural death and deaths from COVID-19, respectively

## Analysis of model (1)

In this section, analysis of equilibria, positivity of solution, basic reproduction number, local stability of the disease-free equilibrium, local stability of the endemic equilibrium as well as parameter estimation and data fitting of model (1) are presented.

### Equilibria of model (1)

The system (1) has the disease-free equilibrium (DFE) at

$$\begin{aligned} X_{o} = & (S_{0} ,E_{0} ,I_{A0} ,I_{S0} ,Q_{0} ,R_{0} ), \\ = & \left( {\frac{\pi }{\mu },0,0,0,0,0} \right), \\ \end{aligned}$$

and the disease-endemic equilibrium (DEE) $\eta_{o} = (S^{*} ,E_{{}}^{*} ,I_{A}^{*} ,I_{S}^{*} ,Q_{{}}^{*} ,R^{*} )$ of (1) is obtained when the right-hand side of (1) is set equal to zero, thus

$$\begin{aligned} & S^{*} = \frac{{\pi \Psi_{2} \Psi_{4} \delta fr_{2} + \Psi_{2} \alpha_{2} \delta f\gamma - \pi \Psi_{3} \Psi_{4} \delta fr_{1} - \pi \Psi_{3} \alpha_{1} \delta f\gamma_{1} - \Psi_{2} \Psi_{3} \Psi_{4} C\delta \mu - \Psi_{2} \Psi_{3} \Psi_{4} C\mu^{2} + \Phi }}{{\delta \mu (\Psi_{2} \Psi_{4} fr_{2} + \Psi_{2} \alpha_{2} f\gamma - \Psi_{3} \Psi_{4} fr_{1} - \Psi_{3} \alpha_{1} f\gamma + \Psi_{3} \Psi_{4} r_{1} + \Psi_{3} \alpha_{1} \gamma }}, \\ & E^{*} = \frac{{C\Psi_{3} \Psi_{2} \Psi_{4} \mu }}{{\delta (\Psi_{2} \Psi_{4} fr_{2} + \Psi_{2} \alpha_{2} f\gamma - \Psi_{3} \Psi_{4} fr_{1} - \Psi_{3} \alpha_{1} f\gamma + \Psi_{3} \Psi_{4} r_{1} + \Psi_{3} \alpha_{1} \gamma )}}, \\ & I_{A}^{*} = \frac{{C\mu \Psi_{3} \Psi_{4} (f - 1)}}{{\Psi_{2} \Psi_{4} fr_{2} + \Psi_{2} \alpha_{2} f\gamma - \Psi_{3} \Psi_{4} fr_{1} - \Psi_{3} \alpha_{1} f\gamma + \Psi_{3} \Psi_{4} r_{1} + \Psi_{3} \alpha_{1} \gamma }}, \\ & I_{S}^{*} = \frac{{C\mu f\Psi_{2} \Psi_{4} }}{{\Psi_{2} \Psi_{4} fr_{2} + \Psi_{2} \alpha_{2} f\gamma - \Psi_{3} \Psi_{4} fr_{1} - \Psi_{3} \alpha_{1} f\gamma + \Psi_{3} \Psi_{4} r_{1} + \Psi_{3} \alpha_{1} \gamma }}, \\ & Q_{{}}^{*} = \frac{{C\mu (\Psi_{2} \alpha_{2} f - \Psi_{3} \alpha_{1} f + \Psi_{3} \alpha_{1} )}}{{\Psi_{2} \Psi_{4} fr_{2} + \Psi_{2} \alpha_{2} f\gamma - \Psi_{3} \Psi_{4} fr_{1} - \Psi_{3} \alpha_{1} f\gamma + \Psi_{3} \Psi_{4} r_{1} + \Psi_{3} \alpha_{1} \gamma }}, \\ & R^{*} = C, \\ \end{aligned}$$

where *C* is positive constant and $$\Psi_{1} = (1 - c_{f} )(1 - c_{d} ), \, \Psi_{2} = \alpha_{1} + \mu + r_{1} , \, \Psi_{3} = \alpha_{2} + \mu + \mu_{1} + r_{2} , \,$$$$\Psi_{4} = \gamma + \mu + \mu_{2} , \, \Phi = \pi \Psi_{3} \Psi_{4} \delta r_{1} + \pi \Psi_{3} \alpha_{1} \delta \gamma .$$

### Positivity of solutions of model (1)

#### Theorem 1

For all time *t* > 0, the solutions of $(S,E,I_{A} ,I_{S} ,Q,R,N)$ of the model (1) are all positive for all $S(0) \ge 0,E(0) \ge 0,I_{A} (0) \ge 0,I_{S} (0) \ge 0,Q(0) \ge 0,R(0) \ge 0$ and $N(0) \ge 0$.

#### *Proof*

From the governing Eq. (1), we obtain

$$\begin{aligned} & \frac{{{\text{d}}N(t)}}{{{\text{d}}t}} \le \pi - \mu N(t), \\ & N(t) \le \frac{\pi }{\mu } + C{\text{e}}^{ - \mu t} , \\ & N(t) \le \frac{\pi }{\mu },\quad {\text{as}}\quad \, t \to \infty . \\ \end{aligned}$$

Hence, the region $\Omega = \left\{ {(S,E,I_{A} ,I_{s} ,Q,R):S + E + I_{A} + I_{s} + Q + R \le \frac{\pi }{\mu }} \right\}$ is positively invariant which implies positivity of solution.$\square$

### Basic reproduction number of model (1)

Using next-generation matrix approach of Heffernan et al. [38], define square matrices *F* and *V* such that $F = \frac{{\partial F_{i} (x_{o} )}}{{\partial x_{j} }}$ and $V = \frac{{\partial V_{i} (x_{o} )}}{{\partial x_{j} }}$ where $F_{i} (x_{o} )$ and $V_{i} (x_{o} )$ are rates of new infections and different ways to move between the compartments $i$, respectively. The compartments $x_{j} (j = 1,2, \ldots ,n \in {\mathbb{N}})$ represent infected compartments for system of Eq. (1), so that

$$\begin{aligned} & F = \left[ {\begin{array}{*{20}c} {\frac{{\Psi_{1} \beta_{E} \pi }}{\mu }} & {\frac{{\Psi_{1} \beta_{IA} \pi }}{\mu }} & {\frac{{\Psi_{1} \beta_{IS} \pi }}{\mu }} & 0 \\ 0 & 0 & 0 & 0 \\ 0 & 0 & 0 & 0 \\ 0 & 0 & 0 & 0 \\ \end{array} } \right], \\ & V = \left[ {\begin{array}{*{20}c} g & 0 & 0 & 0 \\ { - (1 - f)\delta } & {\Psi_{2} } & 0 & 0 \\ { - f\delta } & 0 & {\Psi_{3} } & 0 \\ 0 & { - \alpha_{1} } & { - \alpha_{2} } & {\Psi_{4} } \\ \end{array} } \right], \\ \end{aligned}$$

where $\Psi_{1} = (1 - c_{f} )(1 - c_{d} )$, $\Psi_{2} = \alpha_{1} + \mu + r_{1}$, $\Psi_{3} = \alpha_{2} + \mu + \mu_{1} + r_{2}$, $\Psi_{4} = \gamma + \mu + \mu_{2}$ and $g = f\delta + \left( {1 - f} \right)\delta + \mu$ and then the dominant eigenvalue of the next-generation matrix $G = FV^{ - 1}$ is the basic reproduction number $R_{0}$. Use of Maple software facilitated the process of obtaining inverse of Matrix *V* through a call to “MatrixInverse” command which is capable of computing inverse of a square matrix and can also be used to obtain Moore–Penrose pseudo-inverse of any non-square matrix *A*. The “MatrixInverse” command of Maple adopted for finding inverse of *V* in this study incorporated LU factorization method based on “LUDecomposition” command in Maple 18 which has the capability to solve a matrix with partial pivoting for any matrix A of the form *PA* = *LU* where *L* and *U* are lower and upper triangular matrices and *P* is a permutation matrix. This representation shows that all square matrices can be factorized in this form which is known to be numerically stable and convergent in practice for matrix *V* being considered in this study [39]. Hence, simple matrix multiplication of *F* and inverse of *V* in Maple 18 produced a next-generation matrix G whose dominant eigenvalue is the basic reproduction number

2 $$R_{0} = \frac{{\,\Psi_{1} \,\Psi_{2} \,\delta \,f\beta_{{I_{S} }} \pi + \,\,\Psi_{1} \,\Psi_{2} \,\Psi_{3} \beta_{E} \pi + \,\Psi_{1} \,\Psi_{3} \,\delta \,\beta_{{I_{A} }} \pi - \,\Psi_{1} \,\Psi_{3} \,\delta \,f\beta_{{I_{A} }} \pi }}{{\mu \,g\Psi_{2} \,\Psi_{3} }},$$

for $$\Psi_{1} \,\Psi_{2} \,\delta \,f\beta_{{I_{S} }} \pi + \,\,\Psi_{1} \,\Psi_{2} \,\Psi_{3} \beta_{E} \pi + \,\Psi_{1} \,\Psi_{3} \,\delta \,\beta_{{I_{A} }} \pi > \,\Psi_{1} \,\Psi_{3} \,\delta \,f\beta_{{I_{A} }} \pi$$.

### Local stability of the disease-free equilibrium of (1)

#### Theorem 2

If $R_{0} < 1$, the disease-free equilibrium (DFE) $P_{0}$ of (1) is asymptotically stable but unstable if $R_{0} > 1$.

#### *Proof*

The Jacobian matrix of system (1) for DFE is given by

$$J_{Po} = \left[ {\begin{array}{*{20}c} { - \mu } & { - \frac{{\Psi_{1} \beta_{E} \,\pi \,}}{\mu }} & { - \frac{{\Psi_{1} \,\beta_{IA} \,\pi }}{\mu }} & { - \frac{{\Psi_{1} \,\beta_{IS} \,\pi }}{\mu }} & 0 & 0 \\ 0 & {\frac{{\Psi_{1} \beta_{E} \,\pi \,}}{\mu } - g} & {\frac{{\Psi_{1} \,\beta_{IA} \,\pi }}{\mu }} & {\frac{{\Psi_{1} \,\beta_{IS} \,\pi }}{\mu }} & 0 & 0 \\ 0 & {(1 - f)\delta } & { - \Psi_{2} } & 0 & 0 & 0 \\ 0 & {f\delta } & 0 & { - \Psi_{3} } & 0 & 0 \\ 0 & 0 & {\alpha_{1} } & {\alpha_{2} } & { - \Psi_{4} } & 0 \\ 0 & 0 & {r_{1} } & {r_{2} } & \gamma & { - \mu } \\ \end{array} } \right],$$

where $\Psi_{1} , \, \Psi_{2} , \, \Psi_{3} , \, \Psi_{4}$ and $g$ are defined as in “Basic reproduction number of model (1)” section. The eigenvalues of the matrix $J_{Po}$ are

$$\begin{gathered} \lambda_{1} = - \mu , \, \lambda_{2} = - \mu , \, \lambda_{3} = - \Psi_{4} , \, \lambda_{4} = \frac{{\Psi_{1} \beta_{E} \,\pi \; - g\mu ,}}{\mu }, \, \hfill \\ \lambda_{5} = - \frac{{\Psi_{1} \,\Psi_{2} \beta_{E} \,\pi \, + \,\Psi_{1} \,\delta \,\beta_{IA} \pi - (\,\Psi_{1} \,\delta \,f\beta_{IA} \pi + \Psi_{2} \,g\mu )}}{{\Psi_{1} \beta_{E} \,\pi \, - g\mu }}, \, \hfill \\ \lambda_{6} = - \frac{{\Psi_{1} \,\Psi_{2} \,\delta \,f\beta_{{I_{S} }} \pi + \,\Psi_{1} \,\Psi_{2} \,\Psi_{3} \beta_{E} \,\pi + \,\Psi_{1} \,\Psi_{3} \,\delta \,\beta_{{I_{A} }} \pi - \,(\Psi_{1} \,\Psi_{3} \,\delta \,f\beta_{{I_{A} }} \pi + \Psi_{2} \,\Psi_{3} \,g\mu )}}{{\,\,\Psi_{1} \,\Psi_{2} \beta_{E} \pi + \,\Psi_{1} \,\delta \,\beta_{{I_{A} }} \pi - \,(\Psi_{1} \,\delta \,f\beta_{{I_{A} }} \pi + \Psi_{2} \,g\mu )}}, \hfill \\ \end{gathered}$$

where $\lambda_{i} (i = 1,2,3) < 0$ indicate negative eigenvalues and $\lambda_{i} (i = 4) < 0$ whenever $g\mu > \Psi_{1} \beta_{E} \,\pi$, $\lambda_{i} (i = 5) < 0$ whenever $\Psi_{1} \,\delta \,f\beta_{IA} \pi + \Psi_{2} \,g\mu > \Psi_{1} \,\Psi_{2} \beta_{E} \,\pi \, + \,\Psi_{1} \,\delta \,\beta_{IA} \pi$ and $g\mu > \Psi_{1} \beta_{E} \,\pi$.

Also simplify $\lambda_{6}$ for $R_{0}$ to obtain $$\begin{aligned} & \lambda_{6} = - \frac{{\left( {\frac{{\,\Psi_{1} \,\Psi_{2} \,\delta \,f\beta_{{I_{S} }} \pi + \,\,\Psi_{1} \,\Psi_{2} \,\Psi_{3} \beta_{E} \pi + \,\Psi_{1} \,\Psi_{3} \,\delta \,\beta_{{I_{A} }} \pi - \,\Psi_{1} \,\Psi_{3} \,\delta \,f\beta_{{I_{A} }} \pi }}{{\mu \,g\Psi_{2} \,\Psi_{3} }}} \right)\mu \,g\Psi_{2} \,\Psi_{3} - \Psi_{2} \,\Psi_{3} \,g\mu }}{{\Psi_{1} \,\Psi_{2} \beta_{E} \pi + \,\Psi_{1} \,\delta \,\beta_{{I_{A} }} \pi - \,(\Psi_{1} \,\delta \,f\beta_{{I_{A} }} \pi + \Psi_{2} \,g\mu )}}, \\ & \Rightarrow \quad \, \lambda_{6} = - \frac{{R_{0} \mu \,g\Psi_{2} \,\Psi_{3} - \Psi_{2} \,\Psi_{3} \,g\mu }}{{\Psi_{1} \,\Psi_{2} \beta_{E} \pi + \,\Psi_{1} \,\delta \,\beta_{{I_{A} }} \pi - \,(\Psi_{1} \,\delta \,f\beta_{{I_{A} }} \pi + \Psi_{2} \,g\mu )}}, \\ & \Rightarrow \quad \lambda_{6} = - \frac{{\mu \,g\Psi_{2} \,\Psi_{3} (R_{C} - \,1)}}{{\Psi_{1} \,\Psi_{2} \beta_{E} \pi + \,\Psi_{1} \,\delta \,\beta_{{I_{A} }} \pi - \,(\Psi_{1} \,\delta \,f\beta_{{I_{A} }} \pi + \Psi_{2} \,g\mu )}}, \\ \end{aligned}$$.and $\lambda_{i} (i = 6) < 0$ whenever $R_{0} < 1$ and $\Psi_{1} \,\delta \,f\beta_{{I_{A} }} \pi + \Psi_{2} \,g\mu > \Psi_{1} \,\Psi_{2} \beta_{E} \pi + \,\Psi_{1} \,\delta \,\beta_{{I_{A} }} \pi$, so that the disease-free equilibrium of system (1) is locally asymptotically stable for $R_{0} < 1$, whereas for $R_{0} > 1$ at least one or more eigenvalues are positive and P_0_ is unstable.$\square$

### Local stability of the endemic equilibrium of (1)

#### Theorem 3

The system (1) has endemic equilibrium $P_{1}$ that is locally asymptotically stable for $R_{0} > 1$.

#### *Proof*

Routh–Hurwitz criterion will be used to prove the local stability of the endemic equilibrium of system (1).

Define the Jacobian matrix $J_{P1}$ for DEE by

$$J_{P1} = \left[ {\begin{array}{*{20}c} { - \Psi_{1} \,\Psi_{5} - \mu } & { - \Psi_{1} \,\beta_{E} \,S^{*} } & { - \Psi_{1} \,\beta_{IA} \,S^{*} } & { - \Psi_{1} \,\beta_{IS} \,S^{*} } & 0 & 0 \\ {\Psi_{1} \,\Psi_{5} } & {\Psi_{1} \,\beta_{E} \,S^{*} + g} & {\Psi_{1} \,\beta_{IA} \,S^{*} } & {\Psi_{1} \,\beta_{IS} \,S^{*} } & 0 & 0 \\ 0 & {(1 - f)\delta } & { - \Psi_{2} } & 0 & 0 & 0 \\ 0 & {f\delta } & 0 & { - \Psi_{3} } & 0 & 0 \\ 0 & 0 & {\alpha_{1} } & {\alpha_{2} } & { - \Psi_{4} } & 0 \\ 0 & 0 & {r_{1} } & {r_{2} } & \gamma & { - \mu } \\ \end{array} } \right],$$

where $$- \Psi_{1} = \left( {1 - c_{f} } \right)\left( {1 - c_{d} } \right), - \Psi_{5} = \beta_{E} E^{*} + \beta_{IA} I_{A}^{*} + \beta_{IS} I_{S}^{*} ,g = - f\delta - \left( {1 - f} \right)\delta - \mu$$ let $$T = - \Psi_{1} .\Psi_{5} - \mu , \, B = - \Psi_{1} .(\beta_{E} .S^{*} ), \, H = - \Psi_{1} .(\beta_{IA} .S^{*} ), \, D = - \Psi_{1} .(\beta_{IS} .S^{*} ), \, E = \Psi_{1} .\Psi_{5} , \,$$$$F = \Psi_{1} .(\beta_{E} .S^{*} ) - \delta - \mu , \, W = (1 - f)\delta$$ and the characteristics equation for $$J_{P1}$$ is

3 $$\gamma^{6} + a_{5} \gamma^{5} + a_{4} \gamma^{4} + a_{3} \gamma^{3} + a_{2} \gamma^{2} + a_{1} \gamma^{{}} + a_{0} = 0 \, ,$$

with

$$a_{5} = \mu + \Psi_{4} + \Psi_{3} + \Psi_{2} - F + T,$$

$$\begin{aligned} a_{4} = & \left( {\Psi_{3} + \Psi_{4} - F + T + \mu } \right)\Psi_{2} + \left( {\Psi_{4} - F + T + \mu } \right)\Psi_{3} + \left( { - F + T + \mu } \right)\Psi_{4} + \left( { - F + T} \right)\mu \\ & - \left( D \right)\delta \,f + EB - FT - HW, \\ \end{aligned}$$

$$\begin{aligned} a_{3} = & \left( {\left( {\Psi_{3} + \Psi_{2} - F + T} \right)\mu + \left( {\Psi_{3} - F + T} \right)\Psi_{2} + \left( { - F + T} \right)\Psi_{3} - \left( D \right)\delta \,f + EB - FT - HW} \right)\Psi_{4} \\ & + \left( {\left( {\Psi_{3} - F + T} \right)\Psi_{2} + \left( { - F + T} \right)\Psi_{3} - \left( D \right)\delta \,f + EB - FT - HW} \right)\mu \\ & + \left( {\left( { - F + T} \right)\Psi_{3} - \left( D \right)\delta \,f + EB - FT} \right)\Psi_{2} + \left( {EB - FT - HW} \right)\Psi_{3} \\ & + \left( {E - T} \right)\left( {\left( D \right)\delta \,f + HW} \right), \\ \end{aligned}$$

$$\begin{aligned} a_{2} = & \left( \begin{gathered} \left( {\left( {\Psi_{3} - F + T} \right)\Psi_{2} + \left( { - F + T} \right)\Psi_{3} - \left( D \right)\delta \,f + EB - FT - HW} \right)\mu \hfill \\ + \left( {\left( { - F + T} \right)\Psi_{3} - \left( D \right)\delta \,f + EB - FT} \right)\Psi_{2} + \left( {EB - FT - HW} \right)\Psi_{3} \hfill \\ + \left( {E - T} \right)\left( {\left( D \right)\delta \,f + HW} \right) \hfill \\ \end{gathered} \right)\Psi_{4} \\ & { + }\left( \begin{gathered} \left( {\left( { - F + T} \right)\Psi_{3} - \left( D \right)\delta \,f + EB - FT} \right)\Psi_{2} + \left( {EB - FT - HW} \right)\Psi_{3} \hfill \\ + \left( {E - T} \right)\left( {\left( D \right)\delta \,f + HW} \right) \hfill \\ \end{gathered} \right)\mu \, \\ & + \left( {\left( {EB - FT} \right)\Psi_{3} + \left( D \right)\delta \,f\left( {E - T} \right)} \right)\Psi_{2} + \Psi_{3} \,HW\left( {E - T} \right){, } \\ \end{aligned}$$

$$\begin{aligned} a_{1} = & \left[ \begin{gathered} \left( \begin{gathered} \left( {\left( { - F + T} \right)\Psi_{3} - \left( D \right)\delta \,f + EB - FT} \right)\Psi_{2} + \left( {EB - FT - HW} \right)\Psi_{3} \hfill \\ + \left( {E - T} \right)\left( {\left( D \right)\delta \,f + HW} \right) \hfill \\ \end{gathered} \right)\mu \hfill \\ + \left( {\left( {EB - FT} \right)\Psi_{3} + \left( D \right)\delta \,f\left( {E - T} \right)} \right)\Psi_{2} + \Psi_{3} \,HW\left( {E - T} \right) \hfill \\ \end{gathered} \right]\Psi_{4} \\ & + \left( {\left( {\left( {EB - FT} \right)\Psi_{3} + \left( D \right)\delta \,f\left( {E - T} \right)} \right)\Psi_{2} + \Psi_{3} \,HW\left( {E - T} \right)} \right)\mu \, , \\ \end{aligned}$$

$$a_{0} = \left( {\left( {\left( {EB - FT} \right)\Psi_{3} + \left( D \right)\delta \,f\left( {E - T} \right)} \right)\Psi_{2} + \Psi_{3} \,HW\left( {E - T} \right)} \right)\mu \,\Psi_{4}$$.

From the foregoing, Hurwitz test of necessary but not sufficient condition for a characteristic equation is considered for stability as all coefficients of the characteristics polynomial (3) exist and can be shown to have the same sign. Though not indicated here due to length of the computation, Routh’s stability criterion of necessary and sufficient condition for stability shows that elements of first column of the Routh’s array have the same sign for the characteristic equation represented by (3) under certain conditions using Maple software; consequently all its roots have negative real parts. Hence, Routh–Hurwitz criterion shows that the endemic equilibrium *P*_1_ is locally stable for *R*_0_ > 1.

### Parameter estimation and data fitting

Following the approach used by Adedire and Ndam [27], the parameters that indicate the characteristics of the virus are obtained from the literature. Other parameters that represent non-pharmaceutical infections control strategies such as the use of face masks, social distancing as well as those representing circumstantial effects are estimated from the data obtained from NCDC with modest assumptions using parameter estimation process. The choice of the incubation period follows the research of Chen et al. [37] and Rothana and Byrareddy [40] thus $f = \frac{1}{5.2}$.

The recovery of infected population usually ranges between 3 and 30 days; hence, we made assumption of average recovery period of 15 days for the detected quarantined asymptomatic and symptomatic infectious population with $\gamma = \frac{1}{15}$.

For recovery rates $r_{1}$ and $r_{2}$ from undetected asymptomatic and symptomatic infectious population $I_{A}$ and $I_{S}$, we chose $r_{1} = r_{2}$ representing an average recovery period of 20 days. The assumption of higher recovery rate in the detected quarantined asymptomatic and symptomatic population is based on possibility of special care through administration of certain multivitamins and antibiotics which could significantly boost the immune system of the infected individuals.

Other values of the parameters for non-pharmaceutical control strategies and disease transmission rates together with some initial conditions of model (1) are fitted to the active daily COVID-19 cases and cumulative daily COVID-19 cases with approach of nonlinear least squares technique using Maple software. The total population of Plateau State is estimated as *N*(*t*) = 4,200,442, and the first COVID-19 incidence case in Plateau State was reported on 23 April 2020; the initial conditions of model (1) are estimated as $S(0) \, = \, 4200120$, *R*(0) = 0, *Q*(0) = 1 with $I_{A} (0),I_{S} (0)$ and $E(0)$ estimated from cumulative daily data of COVID-19 obtained from NCDC. It should be noted that the assumption that the data obtained from NCDC are below the actual number of infected cases is adopted due to a few number of tests carried out within the period of this study. Other parameters used in this study are shown in Table 3.

**Table 3:** Base values of parameters used in Eq. (1)

Parameter	Default values	References
$\beta_{E}$	0.01	Data fitted
$\beta_{{I_{A} }}$	0.2	Data fitted
$\beta_{{I_{S} }}$	0.1	Data fitted
$c_{f}$	0.1	[41]
$c_{d}$	0.2	Data fitted
$f$	0.1923	[40]
$\delta$	0.25	Data fitted
$\alpha_{1}$	0.01	Data fitted
$\alpha_{2}$	0.02	Data fitted
$\gamma$	0.067	[37]
$r_{1}$	0.05	Data fitted
$r_{2}$	0.05	Data fitted
$\pi$	0.266e−3	Estimated from data
$\mu$	0.0238e−3	Estimated from data
$\mu_{i} (1,2)$	0.64e−2	Estimated from data
*E*(0)	82	Data fitted
*I*_*A*_ (0)	20	Data fitted
*I*_*S*_ (0)	15	Data fitted

It should also be emphasized here that low initial values are used in parameter estimation process for parameters representing the social distancing and use of face masks. Low parameter assumptions are due to observable low compliance of the population of study to the adoption of strict rules on social distancing and use of face masks. This observable evidence is common among religious gatherings based on their beliefs and also in the market places where rules of social distancing and use of facemasks are not strictly enforced. These initial values representing social distancing and use of facemasks were set low in the fitting of the model Eq. (1) to data using nonlinear least squares method.

## Results and discussion

The objective of this section is to present results and discuss investigation of the impact of non-pharmaceutical control measures such as the use of face masks and social distancing on the transmission dynamics of COVID-19 infectious disease in Plateau State Nigeria. Simulation of model (1) is also carried out in this section using Maple software. The numerical method used to obtain the solution of the proposed infectious disease model (1) is based on Fehlberg fourth–fifth-order Runge–Kutta method with degree four interpolant. It is a robust numerical method of order *O*(*h*^4^) having an error estimator with order *O*(*h*^5^). The numerical method has the capacity to estimate and control errors using higher-order method which permits automatic adaptive step size *h*. Further details on the robustness of the numerical method used can be obtained from [42]. The software was used also to obtain equilibrium points of the proposed governing infectious disease model (1) with constraints set for $S(t) \ge 0,E(t) \ge 0,I_{A} (t) \ge 0,I_{S} (t) \ge 0,Q(t) \ge 0,R(t) \ge 0,t \to \infty$ in line with positivity of solutions of model (1) which has been proved in theorem 1 and seven equilibrium points were obtained.

**Fig. 3:**
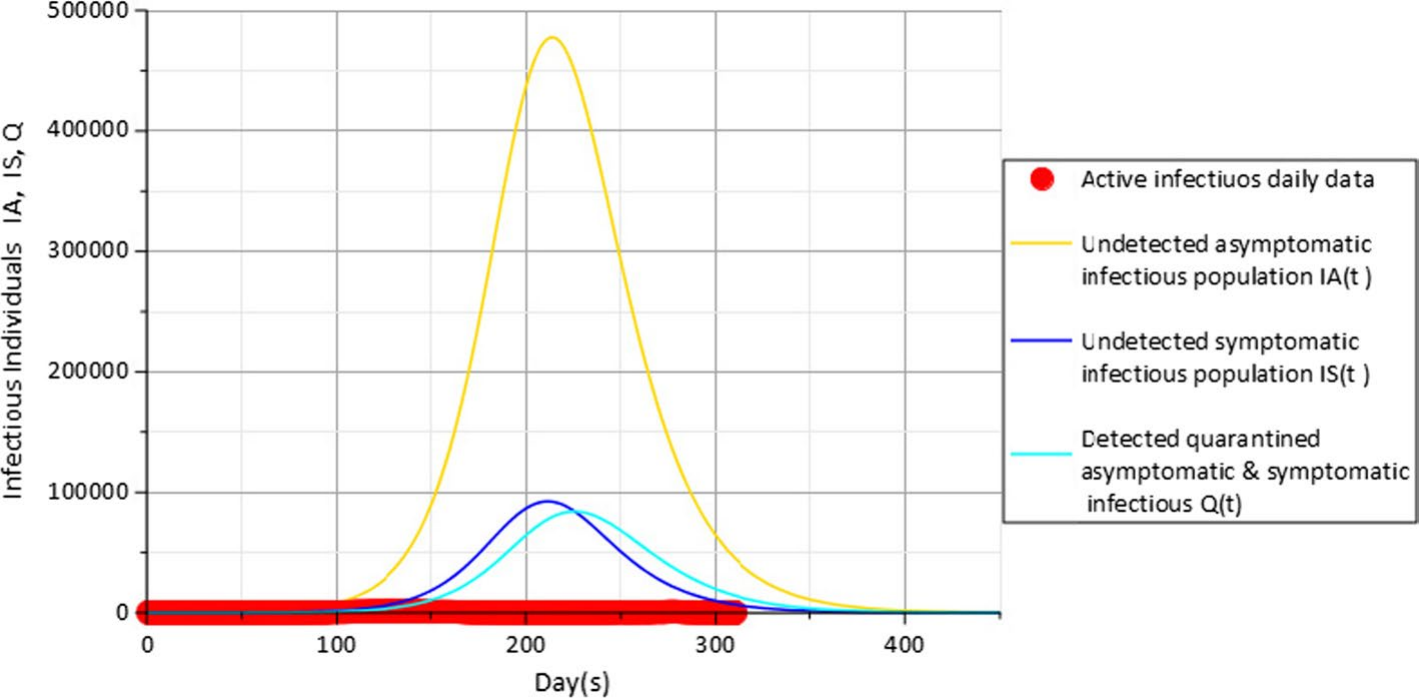
Active daily infectious data (red), $I_{A} (t)$(gold), $I_{S} (t)$(blue) and $Q$(cyan) for $c_{f} = 0.1$ with parameters in Table 3

From the results indicated in Fig. 3, the representation of actual data of the active daily COVID-19 infectious individuals (red) is compared with simulations of undetected asymptomatic population $I_{A}$ (gold), undetected symptomatic population $I_{S}$ (blue) and detected quarantined asymptomatic and symptomatic infectious population Q (cyan) in Plateau State Nigeria.

The values shown in Fig. 3 mean that the model fits well with the actual data and that the peak of the model is at about 213 days, 211 days and 224 days, respectively, for very actively infectious compartments $I_{A}$, $I_{S}$ and *Q* using the parameters $c_{f} = 0.1$ and $c_{d} = 0.2$. The values of the non-pharmaceutical control measures are indicative of low compliance of the Plateau State residents to the use of face masks and social distancing, and Fig. 3 shows that about 476,455 undetected asymptomatic infectious population $I_{A}$(gold), 92,168 undetected symptomatic infectious population $I_{S}$ (blue) and 83,801 detected quarantined asymptomatic and symptomatic population Q(t) (cyan) in Plateau State Nigeria have become infectious at the peak of COVID-19.

**Fig. 4:**
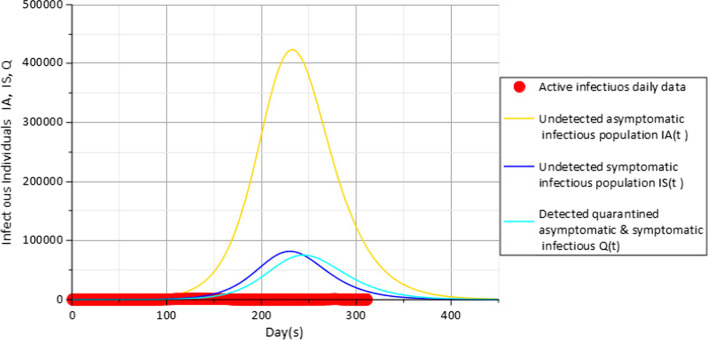
Active daily infectious data (red), $I_{A} (t)$(gold), $I_{S} (t)$(blue) and $Q$(cyan) for $c_{f} = 0.15$ with parameters in Table 3

With non-pharmaceutical control measures $c_{f} = 0.15$, $c_{d} = 0.2$ in Fig. 4, results indicate that the peak shifts to the right in range of about 231 days and 244 days from the date of first incidence case when about 421,619 undetected asymptomatic infectious population $I_{A}$(gold), 81,243 undetected symptomatic infectious population $I_{S}$ (blue) and 74,752 detected quarantined asymptomatic and symptomatic population *Q*(*t*) (cyan) have been infected at peak of the disease.

**Fig. 5:**
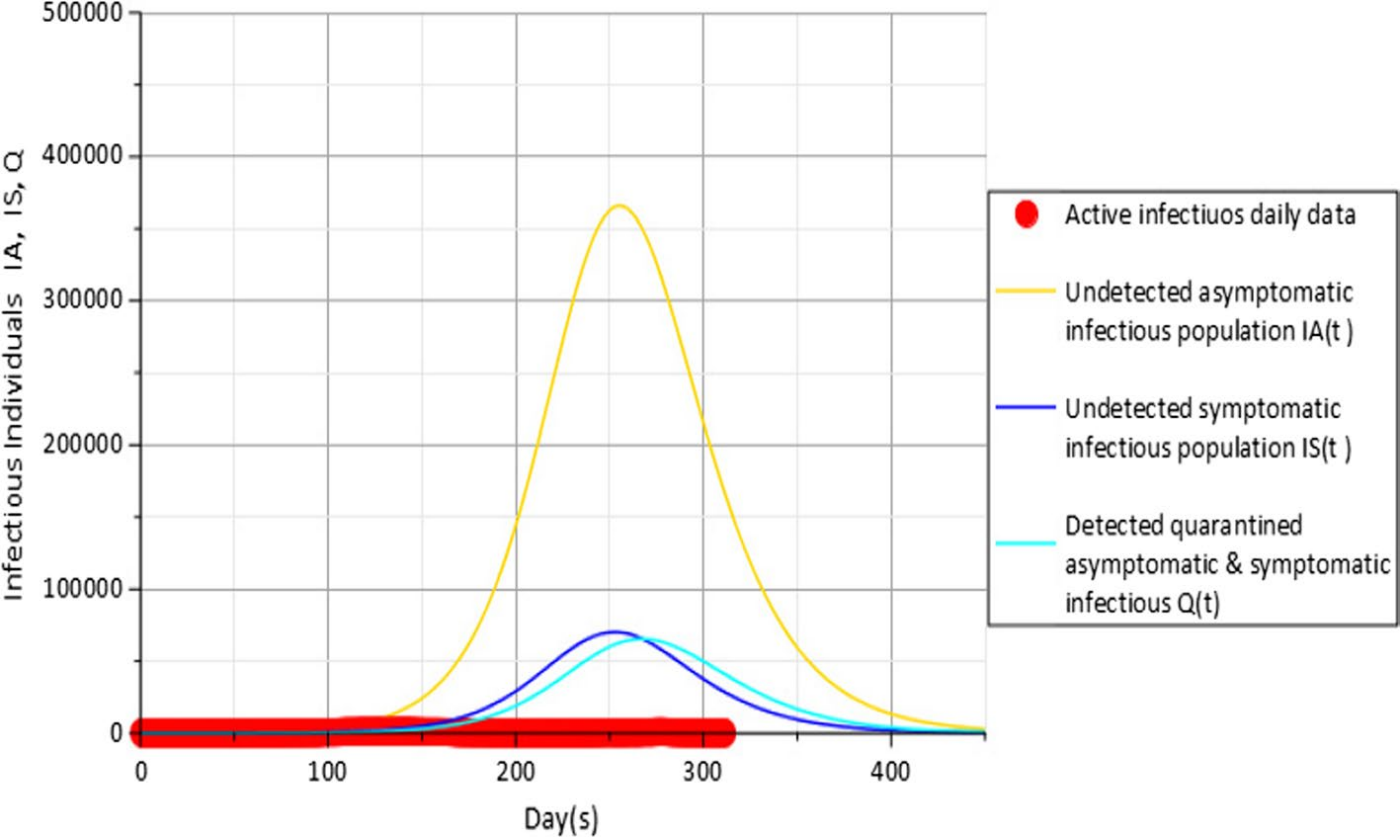
Active daily infectious data (red), $I_{A} (t)$(gold), $I_{S} (t)$(blue) and $Q$(cyan) for $c_{f} = 0.2$ with parameters in Table 3

Results in Fig. 4 also show that a slight improvement in the attitude of the population towards the use of face masks $c_{f}$ from a value of 0.1 to 0.15 representing about 5% increase in the use of face masks could have an impact towards reducing the population of infectious individuals to about 364,864, 70,009 and 65,231 against the 421,619, 81,243 and 74,752 people observed at the peaks in Fig. 3 for the compartments $I_{A} (t)$,$I_{S} (t)$ and $Q(t)$, respectively. Further impact of face masks usage is further observed in Fig. 5 with the value of $c_{f}$ set to 0.2 representing a further 5% increase from the value used in the previous Fig. 4.

Results from Fig. 5 indicate that with $c_{f} = 0.2$, about 364,864, 70,009 and 65,231 individuals from undetected asymptomatic population, undetected symptomatic population and detected quarantined individuals from fully infectious compartments $I_{A} (t)$, $I_{S} (t)$ and *Q*(*t*) have contracted the disease and become infectious in the range of about 252 days and 266 days from the date of first case of COVID-19 in 23 April 2020.

**Fig. 6:**
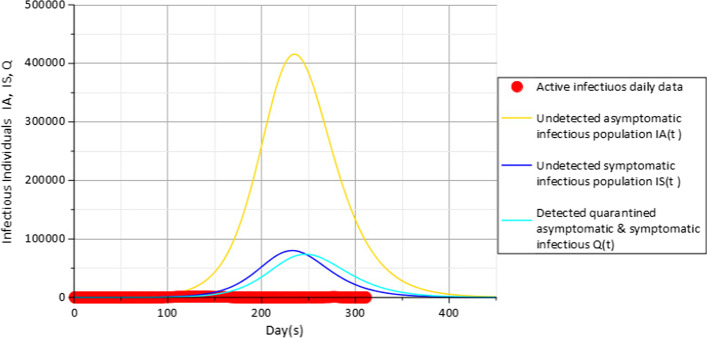
Active daily infectious data (red), $I_{A} (t)$(gold), $I_{S} (t)$(blue) and $Q$(cyan) for $c_{d} = 0.25$ with parameters in Table 3

**Fig. 7:**
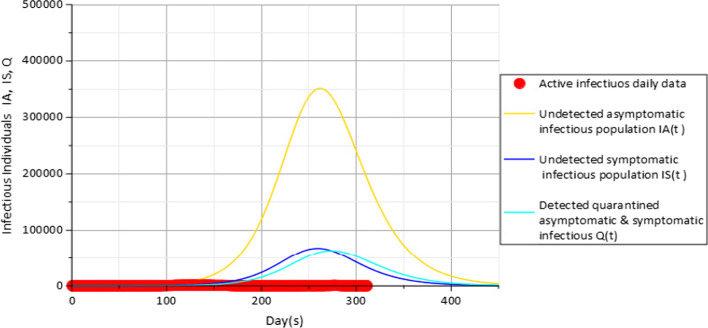
Active daily infectious data (red), $I_{A} (t)$(gold), $I_{S} (t)$(blue) and $Q$(cyan) for $c_{d} = 0.3$ with parameters in Table 3

**Fig. 8:**
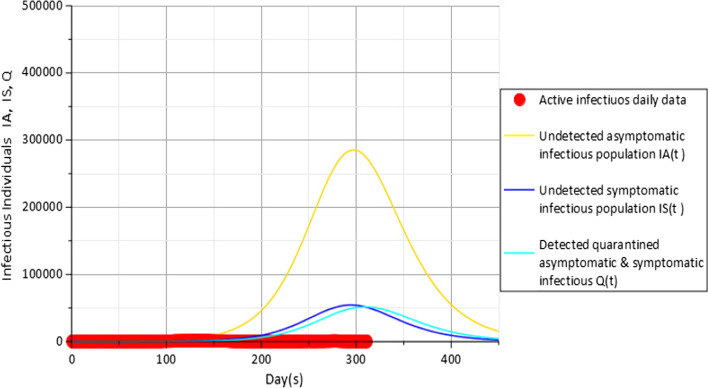
Active daily infectious data (red), $I_{A} (t)$(gold), $I_{S} (t)$(blue) and $Q$(cyan) for $c_{d} = 0.35$ with parameters in Table 3

Effect of social distancing is also investigated as indicated in Figs. 6, 7 and 8. Results from Fig. 6 for social distancing value $c_{d} = 0.25$ suggest about 414,544 undetected asymptomatic infectious population $I_{A}$ (gold), 79,844 undetected symptomatic infectious population $I_{S}$ (blue) and 73,602 detected quarantined asymptomatic and symptomatic population *Q*(*t*) (cyan) at the peak of the COVID-19 incidence. However, a further increase of about 5% in observing social distancing within the population causes a further reduction in the COVID-19 incidence rate to about 350,451 undetected asymptomatic infectious population $I_{A}$(gold), 67,174 undetected symptomatic infectious population $I_{S}$ (blue) and 62,780 detected quarantined asymptomatic and symptomatic population *Q*(*t*) (cyan) as indicated in Fig. 7.

Also from Fig. 8, results show a further reduction in the infectious individuals to about 284,516, 54,279 and 51,406 from 350,451, 67,174 and 62,780 obtained in Fig. 7 when the adherence to social distancing among the susceptible population is improved to a value $c_{d} = 0.35$ for the undetected asymptomatic infectious population $I_{A}$, undetected symptomatic infectious population $I_{S}$ and quarantined asymptomatic and symptomatic infectious population *Q*(*t*), respectively.

**Fig. 9:**
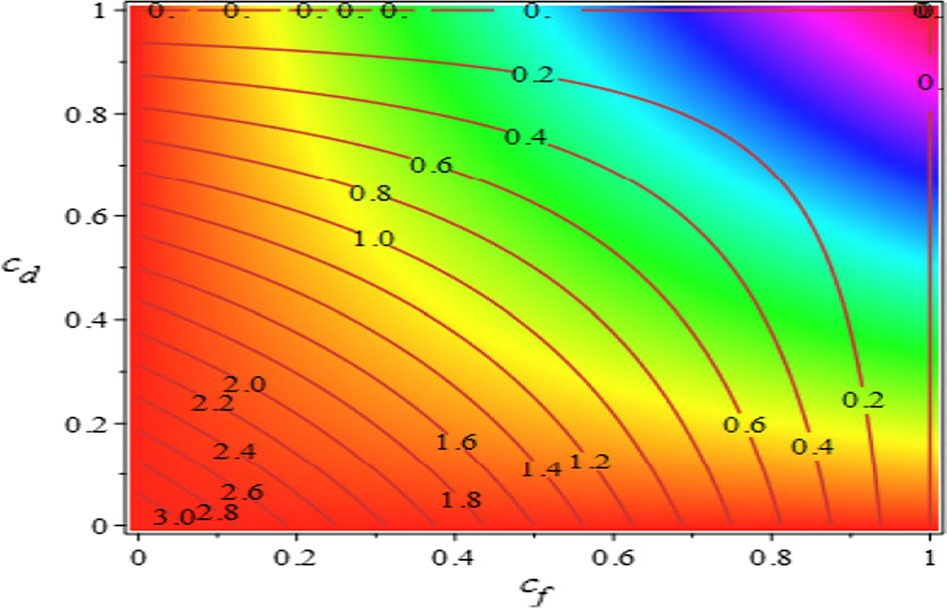
Contour plot of $R_{0}$ for the model (1), as a function of social distancing and face masks

The contour plot of the reproduction number of the model (1) is shown in Fig. 9.

The physical interpretation of basic reproduction number $R_{0}$ lies in its ability to assess the transmissibility of the COVID-19 infections. This means that if $R_{0} < 1$, the outbreak of the disease will die down and the disease can be eradicated. On the other hand, if $R_{0} > 1$, the outbreak of the disease will persist and endemic state will be attained. Also, $R_{0} = 1$ represents the threshold level for determining the persistence level or otherwise of the disease [37, 43].

From Eq. 2, the basic reproduction is 2.3 which is greater than 1 at the baseline values $c_{f} = 0.1$, $c_{d} = 0.2$ and this means that the disease is endemic at the baseline values. Observation from Fig. 9 indicates the possibility of disease persistence with values above 1 at the baseline values. It shows that about 40% in the use of face masks and about 50% of social distancing could bring the basic reproduction number to about 0.96 which is just slightly below 1 and it is within the range necessary for eradication of the COVID-19 in Plateau State. Figure 9 further indicates that if about 50% of the population use face masks and about 60% adopt social distancing, the basic reproduction number gives about 0.64 which further shows possibility that the disease may die out.

**Fig. 10:**
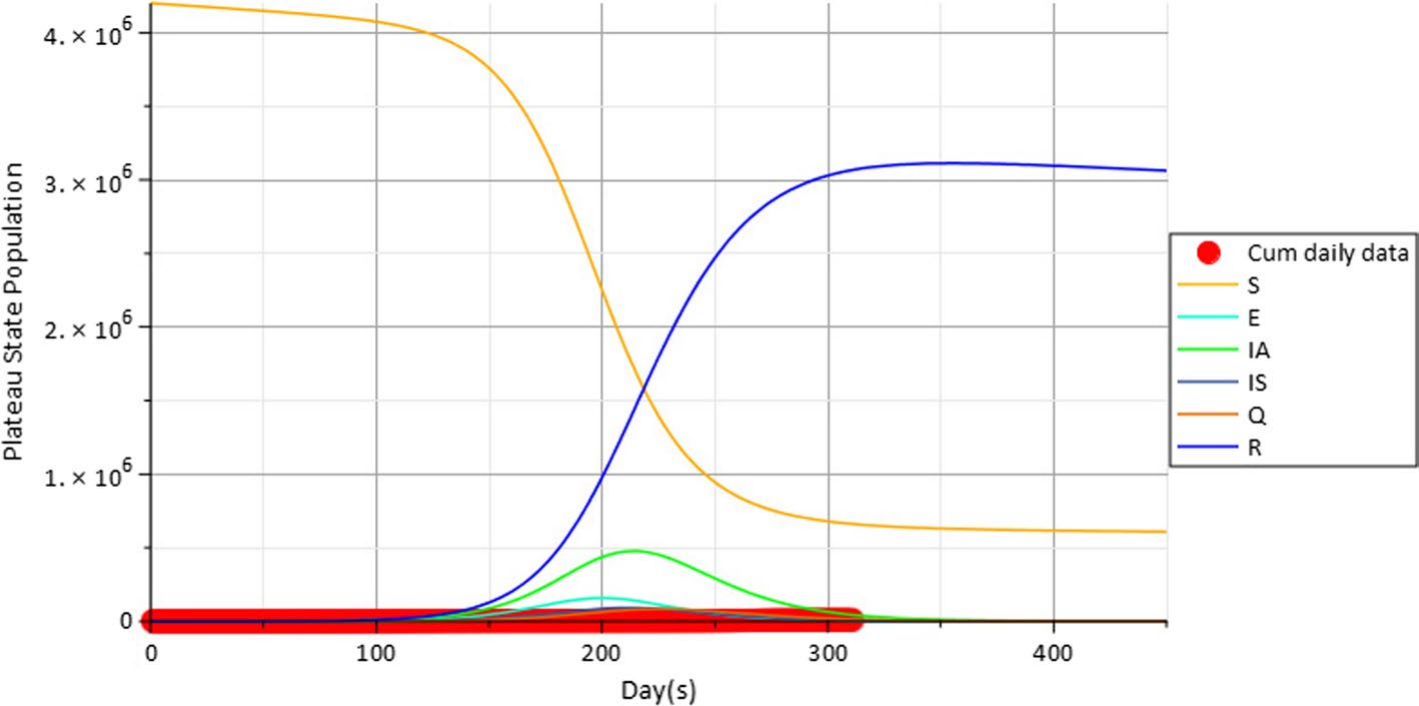
The $S \, E \, I_{A} \, I_{S} \, Q \, R$ and cumulative daily infectious individuals from NCDC (red)

From Fig. 10, the results show $S \, E \, I_{A} \, I_{S} \, Q \, R$ model together with the cumulative daily infectious individuals from NCDC (red). The population dynamics indicate rise in the rate of recovery and decline in the population of the susceptible community. The peak of the infectious classes is also indicated before equilibria states are attained in each of the classes at baseline values.

## Conclusion

In this study, non-pharmaceutical control measures on the spread of COVID-19 in Plateau State Nigeria are examined. The emphasis is on the use of face masks and social distancing. The results indicate that COVID-19 in Plateau State tends to an endemic state at baseline values of control measures. This means that after the proposed infectious disease model (1) of COVID-19 in this study shows prediction of epidemic peak, the level of infection reduces and gradually approaches an endemic state over a period of time. The implication of this is that Plateau State government should be ready to fight the coronavirus for a much longer period than the current transmission wave of the disease.

However, further analysis of results suggests that strict rules on the use of face masks above about 40% of the population and corresponding above 50% adherence to social distancing could as well bring down the basic reproduction number to a value below 1 necessary for disease eradication in Plateau State. Therefore, policymakers in the state need to intensify efforts towards raising the population of users of face masks. Also, compulsory social distancing at public gatherings should be enforced. This study covered a period when non-pharmaceutical control measures were used to curb the spread of COVID-19 in Plateau State. However, vaccination of susceptible class of people has just begun, but the quantity of vaccines available is not yet sufficient to vaccinate majority of the population. Consequently, future researches could incorporate vaccine administration into the model when majority of the population of study have been vaccinated.

## Data Availability

The data sets used for this study are available at public domain of Nigeria Centre for Disease Control [NCDC] under the title: “An update of COVID-19 outbreak in Nigeria”, web link url is at https://ncdc.gov.ng/diseases/sitreps/?cat=14&name=An%20update%20of%20COVID-19%20outbreak%20in%20Nigeria. Accessed 22 April 2021.

## Abbreviations

EOC: Emergency Operations Centres
HBC: Home-based care
COVID-19: Coronavirus disease 2019
NCDC: Nigeria Centre for Disease Control
WHO: World Health Organization
S E I_A_ I_S_ Q R: Susceptible—Latent-Infectious Asymptomatic—Infectious Symptomatic—Quarantined asymptomatic and symptomatic—Recovered
